# Family-related risk factors of obesity among preschool children: results from a series of national epidemiological surveys in China

**DOI:** 10.1186/s12889-015-2265-5

**Published:** 2015-09-19

**Authors:** Xin-Nan Zong, Hui Li, Ya-Qin Zhang

**Affiliations:** Department of Growth and Development, Capital Institute of Pediatrics, No.2 Yabao Road, Chaoyang District, Beijing 100020 China

**Keywords:** Obesity, Risk factor, Family, Behavior, Child, China

## Abstract

**Background:**

Family-based child obesity prevention and control strategy has not yet established in many countries or regions, including China, thus what it needs to do now is to continuously develop and improve the strategies. The purpose of this study were to describe a wider spectrum of risk factors of obesity among preschool children and add to the mounting evidence for further improving suggested intervention measures in future family-based programs.

**Methods:**

Data was collected as part of a series of national epidemiological surveys in childhood conducted in 9 Chinese cities. A population-based, 1:1 matched case–control design was employed to examine risk factors of obesity by means of conditional logistic regression. Obesity was defined as the International Obesity Task Force (IOTF) BMI-for-age cut offs. Eligible subjects were 1234 boys and 610 girls aged 3–7 years in 1996 and 2290 boys and 1008 girls in 2006, including obese and non-obese.

**Results:**

High birth weight, gestational hypertension and parents’ BMI were closely associated with childhood obesity. Breast feeding in the first 4 months was a protective factor in univariate model in 2006 (OR = 0.834, *P* = 0.0234), but the association was not seen in multivariate. Appetite, eating speed, daily time and intensity for outdoor activities, night sleep time, and time for TV viewing were identified statistically by multivariate model. Those children brought up in extended family or mainly raised by their grandparents or lived in high income or low education families might have an increased risk of becoming obese. Parents’ attitudes on weight control of their children significantly differed between obese and non-obese groups.

**Discussion:**

A wider spectrum of risk factors and an empirical aggregation of family-related risk factors are discussed to further improve future family-based child obesity prevention and control strategies. Most of the risk factors identified by this study presented ranked or quantitative characteristics which might be transformed from unhealthy threshold to healthy range by behavior modification. Some variables are likely to interact each other, such as appetite and eating speed, or outdoor activity and TV viewing, or BMI and income, but which needs to be further explored in future surveys.

**Conclusions:**

The family-related risk factors were summarized from our identified risk factors of obesity among preschool children which strongly supported the further development of family-based programs in preschool period.

## Background

Global obesity in children has reached epidemic proportions [1] and increased trend of obesity was also documented in Chinese children over the last decades [2]. Contributing factors to the increases in obesity include a decline in positive health behaviors, such as making healthy dietary choices, engaging in physical activity, and limiting sedentary behaviors. Results on 38 published articles of diet, physical activity, and sedentary behaviors showed support for the role of parenting and physical environmental factors [3]. Family and physical environmental contextual factors related to health behaviors are increasingly the focus of health behavior interventions in line with the bioecological model. Family-based program has been used to treat obesity in youth in some countries, but few programs incorporate a theoretical framework for studying a family systems approach in relation to youth health behavior change [4].

Preschool period is not only the time of the adiposity or body mass index rebound, but also a key link between infancy and school-age period. School-based programs were proved to be effective in reducing obesity for school children [5–7]. School-based and family-involved obesity intervention program is being tested among children aged 10–12 [8]. As schools are an important setting for health promotion for school-aged children, family environment is regarded as a good setting for preschool children, but family-based obesity prevention strategies in childhood were not paid enough attention and not well implemented in practice.

We have noticed that one systematic review showed the strength of evidence was low to support the effectiveness of family-based child obesity prevention programs [9]. The family-based strategy is however still generally on the primary stage and less well-developed. Family-based strategy has not yet established in many countries or regions, including China [10], thus what it needs to do now is to continuously develop and improve the strategies according to own characteristics. In addition, it remains unclear for the practical operability of suggested intervention measures and the compliance of children, parents and families for existing family-based strategies. The purpose of this study were to describe a wider spectrum of risk factors of obesity among preschool children from a series of national epidemiological surveys in China [11] and add to the mounting evidence for further improving suggested measures in future family-based obesity prevention and control programs in childhood.

## Methods

### Data source and subjects

**Fig. 1:**
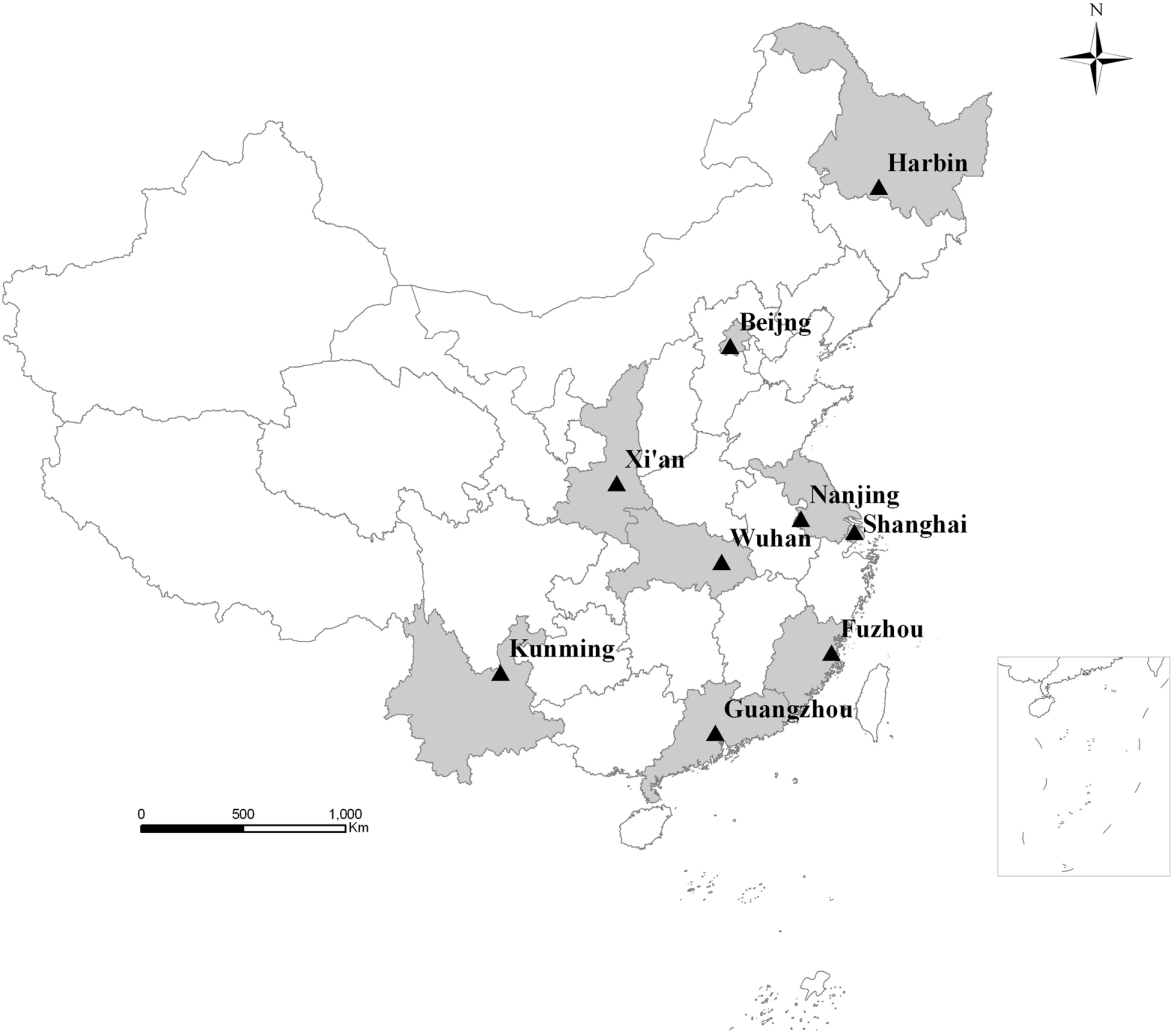
Geographical distribution of the 9 cities (Shaded their corresponding provinces) in China

Data were collected as part of the series of the National Epidemiological Survey on Simple Obesity in Childhood (NESSOC) conducted in 9 Chinese cities (Fig. 1) in 1996 and 2006, respectively. Among the 9 cities, Beijing and Shanghai are municipalities, and the other seven are provincial capital cities: Harbin (Heilongjiang’s provincial capital), Xi’an (Shaanxi), Nanjing (Jiangsu), Wuhan (Hubei), Guangzhou (Guangdong), Fuzhou (Fujian), and Kunming (Yunnan). Multistage stratified cluster sampling was used according to districts and kindergarten. In each city, one or more districts were selected as the study units, and those children aged 3–7 years, living in these study units represented the study subjects. Children at age 3 years or older attended kindergartens and started primary school at 7 years in China. The number of kindergartens was estimated based on the total number of children aged 3–7 years and their age distribution in the selected districts of each city, and the participation rate of the study subjects was not less than 95 % in the selected kindergartens [12]. This study protocol was reviewed and approved by the Ethics Committees of the Capital Institute of Pediatrics. In the series of the NESSOC, consent was verbal, but there is not ethics issue. A written informed parental consent was presented at the head of the questionnaire. Before starting the questionnaire and the physical examination, the staffs of cooperating groups explained adequately the purposes and procedures of the surveys, the risk/discomforts and benefits to parents, guardians, and teachers who understand the potential risks and all the questions involved and voluntarily agree to participate in these surveys.

### Definition, screening and grouping of obesity

Weight and height values of each child documented annually or semiannually through regular physical examination were taken as a crude screening basis in our studies. Current weight and height values were measured in the field sites of kindergartens for determining obesity using a weight-for-height ≥120 % of median of NCSH/WHO [13]. Any extreme values caused by measurement errors could be easily picked out and corrected immediately according to the child’s health record. Secondary or pathological obesity was identified by senior doctors according to “inclusion and exclusion criteria of childhood simple obesity” for separate statistics.

To examine risk factors, the population-based, 1:1 matched case–control design was employed in the series of survey. Due to the variation of potential risk factors among ages and sexes, one identified obese child was matched with one randomly selected non-obese child by sex, age (difference less than half a year) and height (difference within ±3.0 cm) from the same kindergartens.

**Fig. 2:**
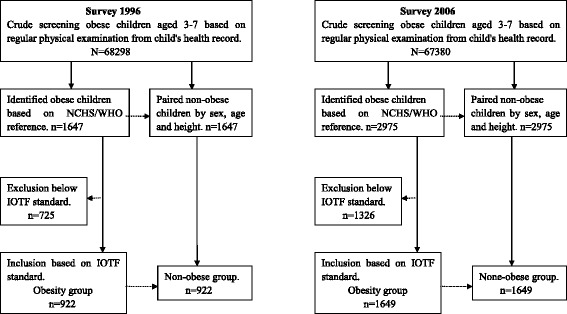
Flow chart of the study population in 1996 and 2006 surveys

In order to make results more likely to be compared, in the present analysis we redefined the obesity using the International Obesity Task Force (IOTF) BMI-for-age cut-off points [14]. Obese children extracted by the IOTF standards from original dataset and their corresponding non-obese children as the subjects of this study were showed in Fig. 2.

### Questionnaire

The questionnaire included child status in early life, maternal condition during pregnancy, dietary habit, behavior and lifestyle, family information/environments. Set of questionnaire items appropriately adjusted in the series of surveys because of the social changes of China. Potential risk factors and confounders of obesity were chosen in the light of previously reported associations, or plausible prior hypotheses.

### Measurement and quality control

Weight and height of all children were measured using unified measuring tools/instruments in a standardized way by specially trained technicians or nurses [15]. The weight was obtained with lever scale to the nearest 0.01 kg with children wearing the lightest vest, shorts or underwear. The height was measured on height board on level ground with bare feet to the nearest 0.1 cm, the back of the head of child, shoulder blades, buttocks and heels all touch the vertical board and a horizontal line from the ear canal to the lower border of the eye socket runs parallel to the baseboard. Errors of weight and height were not more than 0.05 kg and 0.5 cm between two repeated measurements, respectively. Parents could be met in the kindergarten when they picked up their children. Questionnaires involving activity and feeding pattern of the child and parental information such as education level, attitude to obesity and their current weight and height were administered to the parents on the day of investigation. Part of the information about child daily physical activities was reported by kindergarten teachers. The study subgroups of each city collected and checked measurements and questionnaires under the direction of the Beijing Steering Committee that ultimately performed data entry and statistical analysis.

### Statistical analysis

Potential risk factors of obesity were examined using univariate and multivariate conditional logistic regression models. Odds ratio (OR) more or less than 1.00 (*P* <0.05) was considered to be a statistically identified risk factor. Potential interactions were explored between appetite and eating speed, and between outdoor activity and TV viewing, and between parental BMI and income of family. Data were analyzed with SAS version 9.2 (SAS Institute Inc., Cary, NC).

## Results

As outlined in Fig. 2, of the 3294 and 5950 (obese and non-obese) participants based on a weight-for-height ≧120 % of median of the NCHS/WHO reference in the 1996 and 2006 surveys, respectively. The corresponding 1844 and 3298 subjects were extracted in accordance with the IOTF BMI-for-age cut offs. The prevalence of obesity was 1.49 % (boys 1.93 % and girls 1.02 %) in 1996 and 2.64 % (3.47 and 1.74 %) in 2006, with 0.12% point per year of increasing rate (boys 0.15 and girls 0.07). Table 1 presented sample sizes, height and BMI medians (I-III quartile) for obese children and the controls by sex and age in the 1996 and 2006. Comparison of the two groups seems to be not significant difference for height but obviously fatter for BMI in the obese than the controls.

**Table 1:** Sample sizes, median and quartile of height and BMI between obese and control groups in the 1996 and 2006 surveys

	Boys					Girls				
Age (years)	n	Height in cm (M ± Q)		BMI in kg/m^2^ (M ± Q)		n	Height in cm (M ± Q)		BMI in kg/m^2^ (M ± Q)	
		Obese	Control	Obese	Control		Obese	Control	Obese	Control
*Survey 1996*										
3–3.9	76	103.3 ± 7.8	102.1 ± 7.1	20.91 ± 2.07	15.27 ± 2.55	52	104.0 ± 6.7	102.0 ± 7.2	20.29 ± 1.99	16.24 ± 1.35
4–4.9	182	111.5 ± 6.1	109.0 ± 6.0	20.45 ± 1.83	15.30 ± 1.57	106	109.2 ± 5.1	108.3 ± 4.0	20.26 ± 1.41	14.86 ± 1.80
5–5.9	434	117.5 ± 6.6	116.1 ± 6.6	20.79 ± 2.37	15.07 ± 1.58	232	116.0 ± 6.3	115.0 ± 5.8	20.32 ± 1.85	14.89 ± 1.71
6–6.9	542	123.0 ± 5.8	122.0 ± 6.0	21.31 ± 2.08	15.17 ± 1.53	220	122.5 ± 6.2	121.5 ± 6.1	21.35 ± 1.57	14.91 ± 1.83
*Survey 2006*										
3–3.9	177	105.2 ± 4.4	104.5 ± 6.6	20.33 ± 2.17	15.93 ± 1.18	113	103.4 ± 5.1	101.4 ± 6.1	20.32 ± 1.52	15.34 ± 1.30
4–4.9	490	112.7 ± 6.5	111.0 ± 5.6	20.50 ± 1.92	15.35 ± 1.38	278	111.5 ± 6.5	109.3 ± 6.1	20.14 ± 1.28	15.15 ± 1.40
5–5.9	918	119.0 ± 6.8	117.4 ± 5.6	20.59 ± 1.80	15.37 ± 1.43	379	117.5 ± 7.3	116.5 ± 6.9	20.22 ± 1.86	15.14 ± 1.54
6–6.9	705	124.6 ± 6.1	123.5 ± 5.8	21.40 ± 2.24	15.47 ± 1.59	238	123.5 ± 7.5	122.1 ± 5.2	20.79 ± 1.38	14.96 ± 1.41

*BMI* Body mass index, *M* Median, *Q* Quartile (P75-P25)

### Intrauterine and perinatal factors

Tables 2 and 3 showed high birth weight and gestational hypertension were associated with children obesity. Gestational diabetes seemed to have no role in early obesity. From the feeding pattern in the first 4 months, breast feeding was a protective factor in univariate model in 2006 (OR = 0.834, *P* = 0.0234), but the association was not seen in multivariate.

**Table 2:** OR and its 95 % CI of some potential risk factors and confounders of obesity in preschool children using univariate conditional logistic regression

Variables	Factor level of analysis	Survey 1996			Survey 2006		
		N	OR (95 % CI)	P	N	OR (95 % CI)	P
*Intrauterine and perinatal factors*							
Birth weight	<2500, 2500–4000 (ref.), ≧4000	1846			3176		
	2500–4000	1556	1	-	2658	1	-
	<2500	31	1.060 (0.508–2.212)	0.8770	52	0.994 (0.575–1.717)	0.9815
	≧4000	259	1.876 (1.427–2.467)	<0.0001	466	1.880 (1.525–2.319)	<0.0001
Gestational age	<37, 37–42 (ref.), ≧42	1804			3244		
	37–42	1644	1	-	2956	1	-
	<37	85	1.113 (0.715–1.734)	0.6354	134	1.214 (0.853–1.730)	0.2820
	≧42	75	1.642 (1.016–2.654)	0.0436	154	1.117 (0.807–1.546)	0.5030
No of fetuses	1 (ref.), 2 (twins)	-	-	-	3252	0.889 (0.513–1.541)	0.6750
Mode of delivery	Caesarean, vaginal (ref.)	-	-	-	3234	1.381 (1.195–1.595)	<0.0001
Gestational diabetes	No (ref.), yes	1788	0.801 (0.316–2.029)	0.6374	3192	1.647 (0.902–3.009)	0.1011
Gestational hypertension	No (ref.), yes	1798	1.838 (1.231–2.743)	0.0025	3200	2.392 (1.539–3.717)	<0.0001
Feeding patterns in the first 4 months^a^	Breast feeding, formula/milk (ref.)	1846	0.917 (0.731–1.150)	0.4522	3200	0.834 (0.713–0.976)	0.0234
*Dietary habit*							
Appetite	Good, average (ref.), bad	1826			3236		
	Average	447	1	-	1102	1	-
	Good	1335	14.326 (9.651–21.266)	<0.0001	2074	10.995 (8.622–14.02)	<0.0001
	Bad	44	0.519 (0.143–1.883)	0.3123	60	0.209 (0.072–0.602)	0.0038
Eating speed^b^	Fast, average (ref.), slow	1820			3224		
	Average	1126	1	-	1674	1	-
	Fast	444	8.282 (5.739–11.952)	<0.0001	947	7.404 (5.763–9.511)	<0.0001
	Slow	250	0.136 (0.083–0.222)	<0.0001	603	0.227 (0.171–0.302)	<0.0001
Snack	Not at all, occasional, often	1808	1.164 (1.008–1.343)	0.0380	3208	0.948 (0.861–1.044)	0.2789
Vegetable intake	Not at all, occasional, often	1762	0.849 (0.720–1.003)	0.0532	3236	0.869 (0.766–0.986)	0.0292
*Behavior and lifestyle*							
Activity preferred	Outdoor play, TV viewing (ref.), indoor game	1194			-		
	TV viewing	484	1	-	-	-	-
	Outdoor play	479	0.596 (0.446–0.795)	<0.0004	-	-	-
	Indoor game	231	0.876 (0.629–1.221)	0.4337	-	-	-
Daily time for outdoor activity	Time (hr):<half (ref.), half-1, 1–2, ≧2	-			3232		
	<half	-	-	-	367	1	-
	Half-1	-	-	-	1143	0.955 (0.753–1.212)	0.5286
	1-2	-	-	-	1126	0.845 (0.729–0.979)	0.0247
	≧2	-	-	-	596	1.019 (0.773–1.343)	0.8923
Type/intensity of outdoor activity^c^	Running or jumping, walking or cycling, slow walking or sitting (ref.)	1212			3146		
	slow walking or sitting	148	1	-	808	1	-
	Running or jumping	659	0.757 (0.588–0.974)	0.0302	2000	0.725 (0.609–0.863)	<0.0001
	Walking or cycling	405	0.674 (0.351–1.294)	0.1275	338	1.130 (0.869–1.469)	0.3601
Daily time for TV viewing^d^	Time (hr):<half (ref.), half-1, 1–2, ≧2	-			3184		
	<half	-	-	-	447	1	-
	Half-1	-	-	-	1164	1.224 (0.978–1.532)	0.0776
	1–2	-	-	-	1117	1.373 (1.171–1.610)	<0.0001
	≧2	-	-	-	456	1.996 (1.602–2.487)	<0.0001
Daily sleep time.	Unit 0.5 h	1806	0.935 (0.894–0.978)	0.0033	3198	0.942 (0.906–0.981)	0.0035
Night sleep time	Unit 0.5 h	-	-	-	3198	0.890 (0.846–0.936)	<0.0001
*Parents and family-related factors*							
Family type^e^	Nuclear family (ref.), extended, single-parent	-			3242		
	Nuclear family	**-**	-	-	1788	1	-
	Extended	**-**	-	-	1401	1.241 (1.075–1.433)	0.0021
	Single-parent	**-**	-	-	53	0.756 (0.426–1.341)	0.3372
Raising kids mainly by	Parents (ref.), grandparents, or nannies/nursery	1622			3250		
	Parents	1035	1	-	2427	1	-
	Grandparents	399	1.277 (1.010–1.614)	0.0016	702	1.408 (1.181–1.678)	0.0002
	Nannies/nursery	188	0.606 (0.287–1.279)	0.1842	121	1.308 (0.779–2.194)	0.0818
Mother education	College and over, high school and below (ref.)	1794	0.851 (0.667–1.086)	0.1936	3242	0.731 (0.625–0.855)	<0.0001
Father education	College and over, high school and below (ref.)	1798	0.674 (0.521–0.871)	0.0025	3222	0.675 (0.575–0.791)	<0.0001
Family income in local	Upper, middle (ref.), lower	1828			3130		
	Middle	1559	1	-	2968	1	-
	Upper	232	1.040 (0.772–1.402)	0.7955	85	1.546 (0.967–2.473)	0.0668
	Lower	37	1.317 (0.687–2.524)	0.4111	77	1.948 (1.223–3.103)	0.0049
Family income per month	RMB:<1500, 1500–10,000 (ref.), ≧10,000	-			3100		
	1500–10,000	-	-	-	2683	1	-
	<1500	-	-	-	189	1.435 (1.042–1.977)	0.0260
	≧10,000	-	-	-	228	1.430 (1.076–1.901)	0.0152
Father BMI^f^	<24 (ref.), ≧24	1804	2.525 (2.041–3.125)	<0.0001	3152	2.164 (1.866–2.510)	<0.0001
Mother BMI^f^	<24 (ref.), ≧24	1790	4.031 (3.072–5.288)	<0.0001	3192	2.538 (2.075–3.104)	<0.0001
Mother family history of obesity^g^	No (ref.), yes	1826	2.644 (2.110–3.313)	<0.0001	-	-	-
Father family history of obesity^g^	No (ref.), yes	1816	2.640 (2.122–3.284)	<0.0001	-	-	-

*BMI* Body mass index, *OR* Odds ratio, *CI* Confidence interval

^a^Breast feeding including exclusive and partial with >1/2 of total intake

^b^Eating speed, fast (<15 min), average (15–30), slow (≧30)

^c^Running including running, jumping, climbing stair, et al.; sitting meaning not or hardly playing when others did

^d^Time for TV viewing including video game in 2006

^e^Parents-child based nuclear family, three generations based (including grandparents) extended, single-parent based

^f^BMI calculated as weight (kg) divided by height (m) squared based on self-reported weight and height, and overweight in Chinese adults was defined using the cutoffs of 24 kg/m^2^

^g^Obesity history among three generations of parents, including their grandparents, their parents and their siblings, brothers and sisters

**Table 3:** Associations between risk factors and obesity in preschool children using multivariate conditional logistic regression

Variables	Factor level of analysis	Survey 1996		Survey 2006	
		OR (95 % CI)	P	OR (95 % CI)	P
Birth weight	<2500, 2500–4000 (ref.), ≧4000				
	≧4000	-	-	1.609 (1.124–2.303)	0.0094
Gestational hypertension	No (ref.), yes	20.538 (3.453–122.698)	0.0009	2.412 (1.029–5.653)	0.0428
Appetite	Good, average (ref.), bad				
	Good	12.433 (5.562–27.793)	<0.0001	6.266 (4.521–8.686))	<0.0001
Eating speed^a^	Fast, average (ref.), slow				
	Fast	8.039 (3.599–17.960)	<0.0001	4.351 (3.131–6.046)	<0.0001
	Slow	0.173 (0.054–0.555)	0.0032	0.343 (0.225–0.522)	<0.0001
Vegetable intake	Not at all, occasional, often	0.533 (0.334–0.850)	0.0082	-	-
Type/intensity of outdoor activities^b^	Running or jumping, walking or cycling, slow walking or sitting (ref.)				
	Running or jumping	0.191 (0.066–0.847)	0.0022	0.595 (0.445–0.795)	0.0004
Daily time for TV viewing ^c^	Time (hr):<half (ref.), half-1, 1–2, ≧2				
	1–2	-	-	1.339 (1.003–1.789)	0.0477
	≧2	-	-	2.154 (1.455–3.188)	<0.0001
Father education	College and over, high school and below (ref.)	-	-	0.661 (0.494–0.886)	0.0056
Father BMI^d^	<24 (ref.), ≧24	2.326 (1.279–4.232)	0.0057	2.124 (1.634–2.762)	<0.0001
Mother BMI^d^	<24 (ref.), ≧24	2.495 (1.307–4.761)	0.0056	1.529 (1.089–2.146)	0.0142
Raising kids mainly by	Parents (ref.), grandparents, nannies				
	Grandparents	-	-	1.439 (1.050–1.973)	0.0236
Father family history of obesity^e^	No (ref.), yes	1.889 (1.045–3.414)	0.0352	-	-

Numbers contributing to the multivariate analyses: 356 pairs of obese and non-obese children aged 3–7 years in 1996 and 1184 pairs in 2006

Chi-squares of Likelihood ratio and Score for testing global null hypothesis: 300.95 (*P* <0.0001) and 208.52 (*P* <0.0001) for survey 1996 respectively and 851.44 (*P* <0.0001) and 644.02 (*P* <0.0001) for survey 2006 respectively

*BMI* Body mass index, *OR* Odds ratio, *CI* Confidence interval

^a^Eating speed, fast (<15 min), average (15–30), slow (≧30)

^b^Running including running, jumping, climbing stair, et al.; sitting meaning not or hardly playing when others did

^c^Time for TV viewing including video game in 2006

^d^BMI calculated as weight (kg) divided by height (m) squared based on self-reported weight and height, and overweight in Chinese adults was defined using the cutoffs of 24 kg/m^2^

^e^Obesity history among three generations of parents, including their grandparents, their parents and their siblings, brothers and sisters

### Dietary habit, behavior and lifestyle

Appetite and eating speed presented significant positive correlations with obesity. Daily time and type/intensity for outdoor activities were positively connected with obesity in univariate analysis, with their associations of OR = 0.845 (*P* = 0.0247) and 0.725 (*P* <0.0001) respectively, and outdoor activities (running or jumping) also entered multivariate model in 1996 and 2006. Associations of daily sleep time were OR = 0.935 (*P* = 0.0033) in 1996 and 0.942 (*P* = 0.0035) in 2006 according to univariate analysis, and protective effect of night sleep time was OR = 0.890 (*P* <0.0001) in 2006. Daily time for TV viewing was closely related to obesity, with OR = 1.339 (*P* = 0.0477) for 1–2 h level and 2.154 (*P* <0.0001) for≧2 h level in multivariate model in 2006 (Tables 2 and 3).

### Family environment

Compared with parents based nuclear family, those children brought up in extended family (including grandparents) might be at an increased risk of becoming obese. Those children mainly raised by their grandparents might be more susceptible to obesity than those looked after by parents. Higher or lower income families might have a negative effect on weight gain of children, and high income family ultimately entered multivariate logistic regression (Tables 2 and 3).

### Parents’ BMI

Association of children obesity with their parents’ BMI was repeatedly identified in the 1996 and 2006. In addition, the influence of mother and father family history of obesity on children obesity was also observed in the 1996 (Tables 2 and 3).

### Parents’ education and attitude

Parents’ education at college and over might be a protective factor for children obesity, and father education was further screened out by multivariate model in the 2006. In the 1996, 15.7 % parents were not aware of the obesity of their children and 30.0 % parents did not control food and diet intakes for their children in obesity group; only 8.9 % parents helped their children select food in comparison with the proportion of 27.9 % in the controls (*P* <0.01). In the 2006, still 11.2 % parents didn’t realize the obese status of their children for the obesity, and 18.7 % parents believed obesity was a good thing for boys in the obese and the proportion was 14.7 % (*P* <0.01) in the controls.

## Discussion

Not only is obesity increasing worldwide, but no national success stories in obesity prevention and control have been reported over the past decades [16]. Obesity epidemics driven by complex forces require system thinking to conceptualise the causes and to organise evidence needed for action [17]. To the authors’ knowledge, obesity prevention and control for preschool children was not yet paid enough attention in comparison of adults and teenagers. To some extent, data sets of risk factors for childhood obesity are not well established because of the difference of lifestyles, behaviors and social cultures among different nations, countries and regions. Some highly suspected risk factors associated with preschool children obesity have not yet integrated systematically an effective force to counter rapidly rising obesity rates. Childhood obesity prevention research must now move towards identifying how effective intervention components can be embedded within health, education and care systems and achieve long term sustainable impacts [18]. In this study a wider spectrum of risk factors and an empirical aggregation of family-related risk factors are discussed to further improve future family-based child obesity prevention and control strategies.

Genetic factors and intrauterine/perinatal status may influence the susceptibility of individuals to weight gain. The percentage of obesity that could be attributed to genetics varies from 6 to 85 % depending on examined population [19]. Our study demonstrated parental obesity and maternal/paternal obesity affected the weight status of the offsprings which were also observed in rural China [20]. High birth weight and gestational hypertension were considered to be at high risk of future obesity, suggesting the importance of early environments from pregnancy and infancy. Increased risk of birth weight and maternal BMI on early childhood obesity was also confirmed by a cohort study from China [21]. These evidence was consistent with a cohort study on early life risk factors for obesity in childhood in the United Kingdom [22].

Dietary changes towards higher in fats and calories and increasing physical inactivity have been driving progressive increase of weight and epidemic of obesity among Chinese children after the economic transition [23]. Preschool children in Chinese high-income families started to be exposed to those foods with high calorie and high fat, such as Western fast food, fried foods, sweet drinks and so on. Perhaps there are also such some sedentary lifestyles for children in this age as watching TV viewing/video games and taking training courses of outside kindergarten (e.g. foreign languages, drawing and piano). One study from Bangladesh showed limited exercise and high levels of sedentary activities along with having overweight parents more often lead to obesity [24].

There is consensus that changes in lifestyle behaviors are driving the obesity epidemic [25]. Families and parents have profound influence on obesity-related behaviors of their children. Current review has emphasized the role of the home and environment in affecting health behaviors in children and adolescents at high risk for developing chronic disease [3]. Despite observational evidence for the role of parenting in children’s weight-related outcomes emerged, few interventions have been developed that address general parenting in childhood obesity prevention [26]. Our study showed children who were mainly raised by their grandparents were more likely to suffer from obesity, which was also seen in another study from China [27]. Children living in low educational background of parents or families were more likely to be obesity because of a shortage of the knowledge of food selection, energy balance and weight control. Many in China have viewed plumpness as a sign of prosperity and healthy development and accepted the perception “boys should be stronger and bigger”. And because of the impact of the one-child policy, only one child in a family was usually doted upon by their parents and grandparents who are almost given all the treats available with a rising income [28]. Sex difference, higher prevalence of obesity in Chinese boys than girls, reflected the potential impact of ethnicity and socio-culture of different countries or regions. So the perspective about the importance of ethnicity and culture should be taken into consideration [29]. Childhood is an important period of a person for the shape of behaviors, habits and lifestyles which mostly originated from their parents and families. The impact of families on childhood obesity is strongly supported, implying the value of family interventions involving obesity-related behaviors. One recent study concluded the effectiveness of family-based behavioral multi-component intervention for the treatment of overweight and obese children [30].

Most of the risk factors identified by this study presented ranked or quantitative characteristics which might be transformed from unhealthy threshold to healthy range by behavior modification. The present work paves a path for further revising or developing family-based child obesity prevention programs. Future suggesting measures might focus on the amelioration of eating behavior, physical activity, sleep and TV viewing on all fronts to prevent the occurrence of obesity. Our study is in agreement with three major obesity-related behaviors, i.e. dietary intake, physical activity and sleep [31]. Parents played the leading roles in their sharing milieu, and their healthy lifestyles or behavioral habits could be infiltrated to their children. Children in this age are usually enrolled in kindergartens. Earlier studies have indicated that school and family environments are key determinants of energy-balance behaviors in school children. Therefore, kindergartens and families should collaborate with each other to create a better growth environment for the well-being of children.

Some variables are likely to interact each other, such as appetite and eating speed, or outdoor activity and TV viewing, or BMI and income. According to the results of univariate analysis for 2006 data, the interaction between appetite and eating speed was significant for the level of fast eating speed and good appetite, with OR = 30.167 (*P* <0.0001, 95 % CI (19.962-45.588)), average level as control; the interaction of time of outdoor activity and time of TV viewing (as continuous variable) was significant with OR = 1.041 (*P* = 0.0015, 95 % CI (1.016-1.067)); the interactions of income of family and father obesity were significant with OR = 1.479 (*P* <0.0001, 95 % CI (1.356-1.612)) and with mother obesity with OR = 1.522 (*P* <0.0001, 95 % CI (1.354-1.710)). However, these interactions of the aforementioned factors could not enter the multivariate models at the 0.05 level of p value, perhaps their effects need to be further explored in future surveys.

## Conclusions

Health depends more on living conditions, behavior lifestyle and guarantee of medical expenses and the early prevention and control for excessive weight gain is often therefore considered as the most effective way to reduce the risk of overweight and obesity. Although some risk factors identified by this study were also reported in other studies, we observed obvious characteristics of family clustering of risk factors of obesity in the earlier stage of life. A majority of our identified influence factors could be summarized an empirical aggregation of family-related risk factors involving family heredity history, fetal birth weight, parents’ attitude to obesity, eating behavior, physical activity, TV viewing, and raising kids by grandparents. Family-related risk factors, as an integrated concept, would be helpful to promote future family-based prevention and control programs, early detect potential high-risk individuals in early life and enable provision of flexible and personalized interventions based on personal behaviors and needs of the child. According to our results, the family-related risk factors strongly supported the further development of family-based programs in the preschool period. Our study would also add to mounting evidence for future suggesting measures in the process of family-based obesity intervention programs in childhood.

## Abbreviations

BMI: Body mass index
OR: Odds ratio
CI: Confidence interval
NCHS/WHO: National Center for Health Statistics/World Health Organization
IOTF: International Obesity Task Force
